# Overview and Toxicity Assessment of Ultrasound-Assisted Extraction of Natural Ingredients from Plants

**DOI:** 10.3390/foods13193066

**Published:** 2024-09-26

**Authors:** Abayneh Getachew Demesa, Soila Saavala, Marjo Pöysä, Tuomas Koiranen

**Affiliations:** 1 Department of Separation Science, School of Engineering Science, LUT University of Technology, 53850 Lappeenranta, Finland; marjo.poysa@lut.fi (M.P.); tuomas.koiranen@lut.fi (T.K.); 2 Faculty of Technology, LAB University of Applied Sciences, 15210 Lahti, Finland; soila.saavala@lab.fi

**Keywords:** extraction, natural ingredients, phytochemicals, toxicity, ultrasound

## Abstract

In different food technology unit operations, toxicity can be increased due to unwanted side reactions and is typically associated with the increased thermal energy that facilitates the latter. Authorities in food technology have not provided clear guidelines on using ultrasound (US), but they also have not prohibited its use in food processing. In this study, the source materials and ultrasound-assisted reactions reported in the literature were reviewed to investigate potential side reactions in ultrasound-assisted extraction (UAE). Industrial or pilot-scale processes published in the open literature and in industry patents were also examined. UAE is a highly effective extraction method that significantly increases extraction yields. According to the literature, there is no direct evidence of the formation of toxic compounds from natural food ingredients caused by UAE. However, experimental studies are urgently needed to assess the potential accumulation of toxic substances, especially in the case of certain plants.

## 1. Introduction

The term “toxicity” refers to the extent to which a substance is poisonous or capable of causing injury. Human toxicity refers to the capacity of a chemical substance or a mixture of substances to cause harm or adverse effects to human health, ranging from mild irritation to severe illnesssss or even death, depending on the dose, duration, and route of exposure [1]. The safety of food products is controlled at the national level by local legislation and authorities. While there is no global authority to regulate issues concerning food safety, expert committees under the Food and Agriculture Organization of the United Nations (FAO) and The Codex Alimentarius commission provide scientific information on and standardization for food safety matters [2]. Some of the most important local authorities regulating food safety at legislative level are the European Food Safety Authority (EFSA), set up in 2002 by the European Union [3], and the U.S. Food and Drug Administration (FDA) [4]. In the European Union, new food processing methods and technologies need to comply with Regulation (EU) 2015/2283, which defines novel foods as those produced with processes not used in the Union before 15 May 1997, that result in significant changes to a food’s composition, structure, nutritional value, metabolism, or the levels of undesirable substances [5].

UAE has been recognized as a highly efficient extraction technology in food processing that can overcome the drawbacks of conventional extraction methods by virtue of being a non-thermal, fast, higher-energy, and cost-efficient process that can be implemented without toxic solvents. Moreover, ultrasound-based extraction produces concentrated green extracts that are free of any residual solvents, contaminants, or artifacts [6]. Ultrasound can generate cavitation, vibration, crushing, and mixing effects in media, which break cell walls, reduce particle size, and enhance reaction rates through improved mass transfer. This method is effectively applied in natural product extraction, often without altering the structure and function of the extracts [7]. Current applications of power ultrasound, either alone or combined with other technologies, are based on its role as a minimal processing and nonthermal method. It modifies the functional properties of food while preserving its quality attributes, ensuring that flavors, essential nutrients, and vitamins undergo minimal or no changes [8].

In this review, we conducted a preliminary toxicity assessment of ultrasound-assisted natural ingredient extraction from plants in order to evaluate the safety and potential adverse effects of using UAE on food products, focusing on understanding its impacts on nutritional value, bioactive compounds, and overall food safety.

## 2. Effects of Ultrasound-Assisted Extraction on Safety and Toxicity of Food Materials

Ultrasound treatment in food processing offers both advantages and drawbacks concerning food quality. As pointed out by Chemat et al. [9], contrary to common belief, the “natural” state of plant extracts produced with UAE is not a guarantee of product safety. However, the literature does not report any cases of ultrasonic treatment causing toxicity in plant-based food materials. In contrast, non-plant-based products, such as honey, have shown toxicity under extreme conditions, as described by Önür et al. [10]. Negative effects associated with UAE in plant materials are typically linked to extreme processing conditions or improperly optimized parameters, particularly involving rapid temperature increases during processing [11,12,13].

Plants are a rich source of bioactive compounds, including polyphenols, flavonoids, carotenoids, alkaloids, and terpenoids, which contribute to their nutritional, medicinal, and functional properties [14,15]. These compounds play a critical role in human health by acting as antioxidants anti-inflammatory agents and participating in natural defense mechanisms. The extraction of such valuable phytochemicals from plants has garnered significant attention in the field of food processing, pharmaceuticals, and nutraceuticals. UAE has emerged as an effective, eco-friendly technique for extracting these bioactive compounds from various plant materials, offering enhanced efficiency without compromising chemical integrity under normal processing conditions [16,17]. For example, UAE has been shown to improve the extraction of polyphenols, flavonoids, and carotenoids from fruits and vegetables such as grapes, carrots, and spinach [18,19]. Studies have confirmed that UAE enhances the release of these compounds while preserving their safety profiles, with no evidence of toxic by-products under standard extraction conditions [18,19]. This method thus holds great promise for improving sustainable phytochemical extraction, aligning with the growing demand for plant-based ingredients in health and wellness industries [20,21].

### 2.1. Positive Effects of Ultrasound-Assisted Extraction

UAE is widely regarded as an advanced and efficient technique for enhancing the extraction of bioactive compounds from plant-based materials. Its positive effects can be attributed to several key factors.

The use of ultrasound significantly enhances the efficiency of the extraction process, allowing for the recovery of higher yields of valuable compounds such as polyphenols, flavonoids, carotenoids, alkaloids, and terpenoids. It has been observed that UAE enhances both the extraction yields and the functional properties of phytochemicals when properly optimized [22]. These improvements are crucial to maximizing the extraction of bioactive compounds while ensuring safety in the food industry. The intense cavitation caused by ultrasound waves disrupts plant cell walls and membranes, facilitating the release of intracellular compounds into the solvent. Studies have shown that UAE can result in a higher yield of bioactive compounds in a shorter time than conventional extraction methods, such as maceration and Soxhlet extraction [16,17]. This increased efficiency reduces processing time, energy consumption, and solvent usage, making UAE an eco-friendly and cost-effective method for industries focused on plant-based food ingredients.

One of the most significant advantages of UAE is its ability to preserve the chemical integrity of bioactive compounds, such as polyphenols and carotenoids, under standard conditions. Unlike some thermal extraction methods, which can degrade sensitive compounds, UAE operates at relatively low temperatures, minimizing the risk of heat-induced degradation. This preservation of phytochemical integrity is particularly important to maintaining the antioxidant, anti-inflammatory properties, among others, of the extracted compounds, making them suitable for use in functional foods, nutraceuticals, and pharmaceuticals [18,19]. As shown in previous studies, understanding the stability of bioactive compounds extracted by using UAE and their potential antibacterial properties can provide insights into how to better control the extraction outcomes [23].

UAE has demonstrated its effectiveness across a wide variety of plant matrices, including fruits, vegetables, seeds, leaves, and roots. Whether the plant materials are soft fruits such as grapes or fibrous vegetables such as carrots and spinach, UAE can be adapted to extract valuable compounds with minimal modifications to the process. This versatility makes UAE a highly adaptable technique for a range of applications, from food production to natural product extraction in the cosmetics and pharmaceutical industries [20].

Another key advantage of UAE is its ability to improve the bioavailability of extracted compounds. The disruption of plant cell structures and the reduction in particle size enhance the solubility and digestibility of bioactive molecules, which can lead to better absorption in the human body. For example, studies have shown that the molecular weight of polyphenols and polysaccharides can be decreased with UAE, making them more easily absorbed and utilized by the body [11,24]. This is particularly beneficial for the development of functional foods and dietary supplements aimed at improving health outcomes.

In line with the increasing demand for green technologies, UAE offers an alternative to traditional extraction methods, which often rely on high temperatures and large quantities of organic solvents. By utilizing ultrasound waves to facilitate the extraction process, UAE aligns with the principles of green chemistry, reducing the need for toxic solvents and minimizing energy input. This makes UAE a valuable tool in the development of sustainable, eco-friendly extraction processes that meet both regulatory and consumer demands for environmentally responsible practices [17].

### 2.2. Potential Negative Effects of Ultrasound-Assisted Extraction

While UAE offers numerous benefits, potential negative effects are usually observed only under extreme conditions or when the processing parameters (such as ultrasound power, frequency, and duration) are not properly optimized. These negative effects can generally be avoided by properly setting the UAE parameters.

Although UAE is often classified as a non-thermal technique, rapid localized temperature increases can occur during the cavitation process, especially in liquids with poor thermal conductivity or when the processing parameters are not optimized. These temperature spikes, under extreme conditions, can cause the denaturation of proteins and the loss of vitamins and other thermolabile compounds in plant-based food materials [14]. Optimizing temperature control and process duration helps mitigate these effects.

Cavitation-induced free radicals are known to initiate oxidation reactions in food compounds, and this effect becomes more pronounced under extreme UAE conditions or when the power and frequency are too high. While mild oxidation can sometimes yield desirable effects, free radicals can damage proteins, fats, and other bioactive molecules under improperly optimized conditions, leading to off-flavors, texture changes, and unwanted polymers [15,16]. This poses a risk to the nutritional and functional properties of the extracted plant materials if not carefully controlled.

UAE can induce significant changes in the structural properties of plant-based food materials, particularly under high-intensity or long-duration treatments. Proteins may undergo size reduction, changes in rheology, and alterations in the zeta (ζ) potential due to cavitation-induced shear forces [25]. These physical changes can affect the textures of food products, especially in cases where whole plant materials are processed. Non-enzymatic browning and pigment degradation may also occur, leading to undesirable color changes in the final product. However, such effects are largely preventable by performing process optimization [14].

Ultrasound treatment may simultaneously facilitate the extraction of unwanted compounds such as natural toxins. For example, by using UAE, cyanogenic compounds were extracted from the seeds of stone fruits and apple seeds [26], glycoalkaloids were extracted from potato peels [27], and the extractability of quinolizidine alkaloids from lupin was evaluated [28]. However, UAE has only been shown to improve the extractability of these harmful compounds without increasing their toxicity. For instance, while UAE improved the extraction of glycoalkaloids from potato peels, solid-liquid extraction was found to be more effective for this purpose [27]. This highlights the fact that UAE can sometimes enhance the release of toxins, but optimized conditions can help mitigate the associated risks.

As previously stated, there is a need to further study the structural and functional changes caused by ultrasound-assisted extraction (UAE), particularly its effects on the degradation of compounds and the levels of specific bioactive compounds in the extracts [9,29,30]. This remains an area of research critical to optimizing UAE for safe and effective use, ensuring that the process preserves the integrity of the extracted compounds.

## 3. Mechanism of Ultrasound-Assisted Extraction 

**Figure 1:**
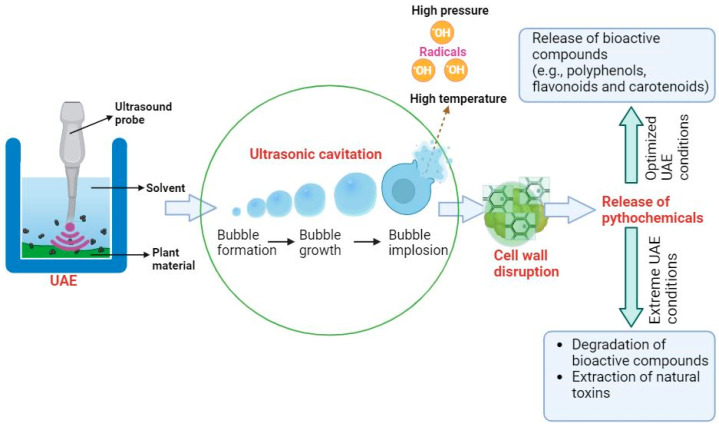
Mechanism of the ultrasound-assisted extraction of bioactive compounds from plant materials. Created with BioRender.com.

Ultrasound-assisted extraction (UAE) is based on acoustic cavitation, a process that occurs when ultrasound waves propagate through a liquid medium, generating alternating high- and low-pressure cycles, leading to the formation, growth, and collapse of microscopic cavitation bubbles. When these bubbles collapse, they produce intense localized shear forces, and high temperatures (up to 5000 K), and pressures (up to 1000 atm), which have a mechanical effect on the plant matrix, disrupting the cell walls and allowing intracellular bioactive compounds, such as polyphenols, flavonoids, carotenoids, and alkaloids, to be released into the solvent [9]. The cavitation process and its effects on the release of these compounds are shown in Figure 1. 

The first stage of UAE involves the formation of cavitation bubbles due to the rapid oscillation of pressure in the solvent. As these bubbles grow and collapse, they create micro-jets and shock waves that physically disrupt the plant-cell structures, enhancing the permeability of the cell membranes and facilitating the release of intracellular phytochemicals [29]. This stage is critical to increasing the solvent’s access to plant tissues and improving mass transfer rates.

Once the plant cell walls are disrupted, the second stage involves the diffusion of bioactive compounds into the solvent, which is facilitated by the mechanical forces generated during cavitation, which in turn, as mentioned above, significantly enhances the rates of mass transfer between the plant material and the solvent. Under optimal UAE conditions, such as moderate ultrasound power, frequency, and temperature, the extracted compounds, including polyphenols, flavonoids, and other antioxidants, are preserved with minimal degradation [9]. Studies have shown that optimizing the parameters can ensure that the bioactive compounds maintain their functional properties during extraction [30].

However, under extreme UAE conditions, such as excessive ultrasound power, prolonged treatment time, or high temperature, the intense mechanical and thermal effects may lead to the degradation or transformation of sensitive phytochemicals. These compounds may break down into simpler molecules or form intermediary compounds through processes such as oxidation or hydrolysis [16]. To mitigate these risks, the optimization of UAE parameters, including power, frequency, and treatment time, is essential to balancing extraction efficiency and the preservation of compound integrity [9].

## 4. Typical Organic Compounds in Plant-Based Ingredients

Plants have complex structures or matrices that should be considered when evaluating the possible toxic effects of UAE. Edible plant parts (e.g., roots, stalks, tubers, bulbs, leaves, stems, fruits, flowers, and seeds) have certain similar characteristics in their compositions even though they form a versatile group, and their properties are dependent on their cultivar, origin, maturity, and storage conditions [31,32]; further, the composition of fruits and other plant parts is strongly dependent on the ripening stage [31,33]. Compounds in fruits and vegetables are formed and regulated through metabolic pathways, where primary metabolism is responsible for the formation of carbohydrates, amino acids, fatty acids, organic acids, hormones, and proteins [33], while compounds that are not essential for the plant, including many phytochemicals, such as terpenoids, phenolic compounds, alkaloids, and glucosinolates, are called secondary metabolites. 

The main component in fruits and vegetables is water (accounting for up to 80–90% of the total weight), followed by polysaccharides, sugars, organic acids, pigments, vitamins, minerals, and flavor compounds [31,32,34]. In most fruits and vegetables, the contents of nitrogen-containing compounds and lipids are low, and the main sugars are the monosaccharides glucose and fructose, as well as the disaccharide sucrose [31], with their ratio varying depending on the type of fruit or vegetable. The main organic acids in fruits are citric acid and L-malic acid. Carotenoids, polyphenols, and chlorophyll are pigment compounds in plant materials [18]; leafy vegetables contain significant amounts of chlorophyll, while flavonoids and carotenoids are typical for most fruits, berries, and vegetables. Flavonoids contain anthocyanins, the main color pigments in fruits and berries, e.g., in grapes [18,19], and betalains are typical for only a few plants, e.g., beetroot. Phenolic acids are present in many plant materials and, similar to vitamins, can act as antioxidants in chemical reactions. A variety of typical chemical compounds in plant materials are listed in Table 1.

Plant materials, e.g., the roots and tubers of vegetables and seeds, have organized structures of storage proteins, triglycerides, starch, and cell wall polysaccharides [31]. Carbohydrates are stored as starch in seeds and lipids as triglycerides in oilseeds. The other main polysaccharides in plants are cellulose, hemicellulose, and pectins. Protein contents are low in fruits and vegetables when seeds serve as storage for these molecules.

A wide variety of compounds are formed via chemical reactions during plant metabolism and the processing of plant materials. Plants have complex biosynthetic pathways and molecular regulation mechanisms, which cause several volatile flavor compounds to form, e.g., aldehydes, alcohols, ketones, and terpenes [34]. US treatment has been observed to promote the formation of Maillard reaction products such as pyrazines and 5-hydroxymethyl-2-furaldehyde (HMF) [35,36].

## 5. Ultrasound-Assisted Extraction Applications

The literature describes a wide variety of laboratory-scale applications of UAE from various sources for products intended for human consumption. Ultrasound has been recognized for its potential industrial applications in the phyto-pharmaceutical extraction industry for a wide range of herbal extracts [30]. Potential extraction products include bioactive compounds (polyphenols, anthocyanins, tartaric acid, aroma compounds, polysaccharides, etc.); oils from soybeans, caraway seeds, *Jatropha curcas* L., almond, and apricot seeds [18]; and pectin, dietary fibers, and oils from fruit and vegetable processing byproducts [37]. Ultrasound has also been applied in the extraction of hemp seed oil (*Cannabis sativa* L.) [38], olive oil [39], and flaxseed oil [40]. The extraction of bioactive compounds from marine algae [41] and of plant-based proteins [25] has been studied. Ultrasound has been effectively used to extract various classes of compounds, including aromas, polyphenols, organic substances, and minerals, from a wide range of matrices [14]. The application of ultrasound in the extraction of color pigments of plant origin has been reviewed [37,42], and the possibilities of sonication in the optimization of cold brew coffee production [43] and winemaking [44] have been studied. Some specific applications previously reported include starch extraction from mango kernels [45] and the extraction of β-asarone from sweet flag (*Acorus calamus*) rhizome [46].

There are only a few literature findings on large-scale processes utilizing ultrasonic-assisted extraction, a gap in research also recognized by Shen et al. [11] in their comprehensive review. Ultrasonic extraction was tested in olive oil production on a production scale in 2014 [47], and Hielscher Ultrasonics created a production-scale process for ultrasonic-extracted olive oil [48]. Pingret et al. [49] identified three companies applying UAE commercially in aromatic herb extraction; the production of food, pharmaceutical additives, and alcoholic drinks; and the extraction of thermolabile compounds with alimentary and cosmetic applications. Tamminen et al. [50] reported scaling up a continuous, ultrasound-assisted extractor for plant extracts. A Finnish company, Kääpä Biotech, reported using UAE in the fungal extraction of bioactive-enriched consumer products [51].

A search in the Espacenet database with the terms “ultrasonic-assisted extraction” and “ultrasound-assisted extraction” in the title yielded a total of 106 patents. Among these, 68 patents were directly related to methods or technologies for extracting various compounds from plants or fungal by using UAE. A significant portion of these patents originated from China and focused on the extraction of bioactive compounds commonly used in traditional Chinese medicine. These bioactive compounds are derived from plant and fungal materials and are processed by using various UAE techniques to improve efficiency and yield. The full list of these patents, including details on the specific compounds and extraction methods, can be found in Table S1, located in the Supplementary Material section.

## 6. Reaction Intensification in Ultrasound-Assisted Organic Synthesis and Chemical Reactions

Ultrasound can be used in the intensification of organic synthesis reactions and in the enhancement of extraction efficiency. For food matrices, it is mainly used for the improved extraction or removal of components. A high-intensity ultrasound field has been found to increase yields and drastically reduce reaction times in certain organic reaction systems [52], which is due to the cavitation and micro-mixing intensification induced by ultrasound. In the case of homogeneous-phase reactions, cavitation mainly produces radicals that influence reaction progression, while heterogeneous reactions also involve solid particles that are affected by ultrasound-based cavitation. In this case, the reactions taking place on solid catalysts’ surfaces are influenced by ultrasound cavitation and micro-mixing [53,54]. In this study, we limited the investigation of the effect of ultrasound in chemical reactions to high-power ultrasound (frequencies of 20–40 kHz), as it has the highest effect on extraction enhancement. Classical reactions are included in Table 2, and in Table 3, more specific reactions in food matrices are listed. 

**Table 2:** Ultrasound-intensified organic synthesis reactions.

Reaction Type	Starting Materials	Reaction Conditions	US-Effect	Reference
Mannich-type reactions	Aldehydes, ketones, and amine	Alkaline EtOH-water/methyl sulfonate, 20 °C, nominal US power of 600 W, lab-scale	18 h for high-speed stirring to achieve 88% yield; US-assisted process achieved 95% yield in only 1.5 h	Zeng et al. [55]
Aza- Michael reactions	Amine (imidazole) + carbene	Water, +20 °C	30 min for 92% yield with conventional stirring; US method reduced time to 5 min and increased yield to 98%	Yao and Pan [56]; Bandyopadhyay et al. [57]
Stille coupling reactions	Organotin + halide	Palladium-catalyst	Reaction time reduced from 20 h to 30 min; US significantly enhanced reaction rate, achieving 97% yield	Yao and Pan [56]
Homogeneous acid-catalyzed transesterification	Fatty acid + alcohol	Strong acid catalyst	95.5% yield achieved in 2 h by using sulfuryl chloride as catalyst with US	Yao and Pan [56]
Interesterification	Fatty acids migration from one position to another	Normal temperature	Fast and efficient approach for biodiesel production with significant reduction in reaction time and enzyme loading	Yao and Pan [56]
Heterogeneous acid-catalyzed transesterification	Fatty acids	Catalysts: alumina/zirconia/Ti/ZnO	High yields of esterification of oleic acid under ultrasonic irradiation	Yao and Pan [56]
Synthesis of glycerol carbonate catalyzed by lipase	Glycerol carbonate, DMC	US power of 0–200 W, US frequency of 25 and 40 kHz, Enzyme: Lipase (Novozym 435)	Ultrasound achieved 99.75% limiting equilibrium conversion, saving 10 h compared to stirring	Yao and Pan [56]
Lutein disuccinate synthesis	Succinic anhydride	Reaction in DCM, with TEA as catalyst	Limiting equilibrium conversion iincreadsed from 56% in 12 h with conventional method to 80% in 2 h with US	Yao and Pan [56]
Multi-component synthesis	Malononitrile, 3,5-dione, and aromatic aldehydes	Iodine catalyst	US-assisted synthesis achieved 95% yield in 10 min; more efficient than conventional approach	Yao and Pan [56]
Glycine assisted multi-component reaction	Malononitrile, resorcinol, and aromatic aldehydes	Reaction in H_2_O and glycine as organocatalyst, at 30 °C, reaction time of 9–45 min	Product purity ranged from 88 to 96% under US conditions	Cravotto et al. [58]

**Table 3:** Food matrix-based chemical reactions under ultrasonic field.

Reaction Type	Starting Materials	Reaction Conditions	US-Effect	Reference
Oxidation reactions	Water + any oxidative hydrocarbon	Water, +20 °C	Conventional heating at 90 °C gave 97% yield in 60 min; US-assisted process achieved 98% yield in 15 min	Chatel et al. [59]
	Oxidation reactions (sonication of water leads to the formation of radicals)	US 24 kHz for 120 min	Cephalexin amino acid degraded	Babu et al. [60]
Protein degradation	Soybean protein isolate and egg white protein	Water, <35 °C	Formation of sulfhydryl groups in processing times of 20–40 min and 5–30 min, 20 kHz, high-power ultrasound (60 W/cm²)	Ren et al. [61]
Maillard reaction	Glucose + lysine	Water, +60 °C	Slight increase in lactulose after 15 min during lactose isomerization compared with conventional heating	Corzo-Martinez et al. [62]
	Glycine + maltose	Water, +50 °C	Greater processing times (>20 min) promoted pyrazines production (harmful but not toxic)	Guan et al. [35]
Enzymatic hydrolysis reactions	Lard hydrolysis	5 min US in water, with lipase enzyme	Nearly 100% hydrolysis achieved under US conditions	Cordova et al. [63]
	Starch hydrolysis	Glucoamylase, high US power density of 2–14 kW/L, 35–75 °C, 10–50 min	Significant improvement in starch hydrolysis under US field	Cordova et al. [63]

According to Yu et al. [64], the use of sound waves is considered a safe, nontoxic, and environmentally friendly processing method, which gives ultrasound a major advantage over other emerging techniques. Most of the reactions listed in Table 2 are not relevant in ultrasound-assisted extraction from biobased food matrices, but transesterification reactions can take place in the case of fatty acids. Among the studies in Table 3, in a few reports, degradation was identified as a potential issue and the formation of harmful pyrazines was reported by Guan et al. [35]. However, only Önür et al. [10] reported the formation of a toxic component, 5-hydroxy methyl furfural, during the ultrasound treatment of honey at 60 °C for 2 h.

Aldehydes, ketones, and amines can undergo Mannich-type reactions in an alkaline ethanol–water mixture, as shown in Table 2. Food matrices and solutions rarely have an alkaline pH, and ethanol may be produced during fermentation, but methyl sulfonate is not typically present in foods. In theory, sugars, volatile flavor compounds, and Maillard reaction compounds can be reactive under suitable conditions. Free amines, which may be reactive via aza–Michael reactions, could be available, e.g., in fruits, but carbene is not present in foods. The most probable reactions occurring in foods during ultrasound-assisted extractions involve fatty acids. Apart from oilseeds and certain fruits, plant materials generally have relatively low lipid contents. Esterification reactions were reported in the studies summarized in Table 2. Aromatic aldehydes, which can occur in spices, such as cinnamaldehyde in cinnamon and vanillin in vanilla beans, could potentially participate in reactions under specific conditions. The assessment shown in Table 2 confirms that the known ultrasound-assisted reactions require reagents that are not typically found in food solutions.

Possible ultrasound-intensified reactions for food matrices are described in Table 3. It is known that the formation of hydroxyl radicals occurs both in conventional heating and in ultrasound-assisted processes [65,66] and that rancidity in foods caused by the oxidation of fatty acids [31]. The denaturation of proteins and changes in their conformation are also typically caused by heating and have been reported to occur during ultrasound treatment [66,67]. The latter has further been reported to change the primary structure of proteins [66,67] and polysaccharides [11,45,67] and to cause size reductions for fat crystals [36] and legume proteins [66]. In addition, the reduction in the molecular weight of polyphenols due to the possible breakdown of glycosidic bonds has been reported [11]. Ultrasound treatment may alter protein conformation and structure, leading to changes in sulfhydryl groups and the exposure of hydrophobic groups, which can result in protein denaturation. Ultrasound treatment has also been reported to break down starch chains and amylopectin in mango seed kernels [45], and the depolymerization of homo- and heteropolysaccharides (dextran, starch, pectin, guar gum, and xanthan) may also occur [67]. These changes could result in increased reactivity, e.g., due to exposed proteins sidechains and polysaccharide hydrolysis products, but there are no US-related studies available.

The Maillard reaction simultaneously creates desirable flavor compounds and causes the formation of possible harmful compounds. Reducing sugars and certain amino acids, such as lysine, are prone to react when heated [31]. While uultrasound treatment has been reported to promote the formation of Maillard reaction products [36], it is not the only technique with which new compounds are produced when food materials are processed. To conclude, the ultrasound-intensified reactions in Table 3 do not show an elevated risk of ultrasound-induced toxicity in comparison with conventional food processing methods. Nevertheless, high intensity or excessive ultrasound power and long duration should be avoided, with the aim of optimizing the treatment parameters to minimize process-induced changes.

## 7. Conclusions and Future Directions

Ultrasound-assisted extraction (UAE) is a safe and effective technology for extracting natural, plant-based ingredients, with a strong track record of preserving the nutritional value and bioactive components of food extracts. This method has also demonstrated the ability to effectively reduce toxicity by aiding in the removal of natural toxins such as cyanogenic compounds, glycoalkaloids, and alkaloids, which may be present in plant materials. These toxins can enter the food chain through natural biological processes or environmental contamination. While UAE enhances the extractability of these compounds, its ability to prevent increases in toxicity makes it an excellent tool for improving food safety.

Optimizing factors such as ultrasound frequency, output power, temperature, pH, and treatment time is crucial for minimizing any negative impacts on food properties, including color and the contents of phenolics, vitamins, and bioactive components. Under optimized conditions, the negative effects on these properties are generally minimal. Although rare, higher concentrations of natural toxins may be extracted in certain cases, emphasizing the importance of carefully adjusting the processing parameters to mitigate risks. UAE has also been shown to have minimal impact on food texture and structure when the parameters are carefully controlled, preserving the overall quality of the final product.

In addition to its ability to enhance the safety and quality of plant-based extracts, UAE contributes to sustainable food processing by reducing solvent use and energy consumption, making it a green and eco-friendly alternative to conventional extraction methods. The overall evidence strongly suggests that UAE, when optimized, can be utilized to extract natural ingredients efficiently and safely, ensuring food safety while preserving bioactive compounds.

Future research should focus on further refining the UAE processing parameters to improve the safety and quality of the extracts, particularly in minimizing the risks associated with the extraction of natural toxins. Investigating the formation of intermediate compounds, the potential for the degradation of bioactive components, and the biological effects of UAE-treated compounds will be crucial to advancing the application of UAE. Based on continuous process optimization, UAE can remain a reliable and sustainable method for producing high-quality natural extracts, enhancing both food safety and nutritional value.

## Figures and Tables

**Table 1:** Typical chemical compounds in plant materials.

Group	Compound	Source	Reference
Mono- and disaccharides	Sucrose, fructose, and glucose	Apple, watermelon, strawberry, pear, peach, and tomato	Xu et al. [34]
Organic acids	Malic acid and citric acid	Apple, watermelon, strawberry, pear, and peach	Xu et al. [34]
	Tartaric acid	Grape, pear, and apple	Xu et al. [34]; Vilkhu et al. [19]
	Aascorbic acid	Orange, berries, and potato	Jideani et al. [32]; Rodríguez Garcia and Raghavan [20]
Carotenoids	Alpha-carotene, beta-carotene, and lycopene	Apricot, carrot, mango, orange, and tomato	Jideani et al. [32]; Rodríguez Garcia and Raghavan [20]; Kumar et al. [18]
	Xanthophylls (lutein, neoxanthin, and zeaxanthin)	Broccoli, spinach, and lettuce	Jideani et al. [32]; Rodríguez Garcia and Raghavan [20]; Kumar et al. [18]
Chlorophyll	Chlorophyll a and b	Leafy vegetables	Kumar et al. [18]
Betalains	Bbetacyanin and betaxanthin	Beetroot	Jideani et al. [32]
Flavonoid polyphenols	Flavonols, flavanols, and anthocyanins	Apple, banana, carrot, grape, berries, and broccoli	Jideani et al. [32]; Vilkhu et al. [19]
Non-flavonoid polyphenols	Phenolic acids, benzoic acids, and cinnamic acids	Apple, apricot, banana, berries, grape, and cabbage	Jideani et al. [32]
Ttannin polyphenols	Proanthocyanidins and soluble tannins	Broccoli	Jideani et al. [32]
Fatty acids	Stearic acid, oleic acid, linoleic acid, and linolenic acid	Olive, palm fruit, oilseeds, avocado, and herbs	Rodríguez Garcia and Raghavan [20]; Gouda et al. [21]
Terpenes	Limonene	Carrot, orange, caraway seeds, and herbs	Jideani et al. [32]; Gouda et al. [21]
Glucosinolates	Isothiocyanates	Broccoli, cabbage, cauliflower, and leaves	Jideani et al. [32]; Kumar et al. [18]
Steroid alkaloids	Alpha-solanine	Potato and eggplant	Xu et al. [34]

## Data Availability

No new data were created or analyzed in this study. Data sharing is not applicable to this article.

## Supplementary Materials

The following supporting information can be downloaded at 361 https://www.mdpi.com/article/10.3390/foods13193066/s1: Table S1: Patents on ultrasonic-assisted extraction methods.
