# Supplementary material for: Overview and Toxicity Assessment of Ultrasound-Assisted Extraction of Natural Ingredients from Plants
Source: Foods. 2024 Sep 26;13(19):3066. doi: 10.3390/foods13193066 (PMC11476364; doi:10.3390/foods13193066)
Supplement: Supplementary file 1 [file foods-13-03066-s001.zip › foods-3195932-supplementary.pdf]

Table S1: Patents on ultrasonic-assisted extraction methods

	Title	Inventors	Applicants	Publication Number	Publication Date
1	Ultrasonic-assisted extraction method of grifolan	Dai Lu	Harbin Moyi Biotechnology Co. Ltd.	CN104387492A	2015-03-04
2	Ultrasonic-assisted extraction method of flaxseed protein	Wang Yong, Zhang Zhen, Ge Jiajia, Deng Fenggui	Guangzhou Lizhong Biological Tech Co. Ltd.	CN108892705A	2018-11-27
3	Method for ultrasonic-assisted extraction of lentinan	Cai Wen, Duan Zhenhua, Zhu Dongjian, Shuai Liang	University of Hezhou	CN110655592A	2020-01-07
4	Ultrasonic-assisted extraction method of asarum polysaccharide	Yan Peizheng, Zhang Jingyi, Mao Beibei, Liu Yan, Chen Yufan, Zhao Mingzhe, Wang Bin, Li Xiangyang, Liu Yihan	University of Shandong Traditional Chinese Medicine	CN116515004A	2023-08-01
5	Ultrasonic-assisted extraction technology of macadamia nut polypeptide	Chen Jinji	Maoming Dianbai Jiule Food Co. Ltd.	CN107821730A	2018-03-23
6	Method for ultrasonic-assisted extraction of ginger oil	Zeng Caibing	Guangxi Xilin Nongben Biotechnology Co. Ltd.	CN109022122A	2018-12-18
7	Technology for ultrasonic-assisted extraction of valerian polysaccharide	Chen Shixue, Lu Daowang, Lu Zhongying, Yao Yuanyong, Xing Mingming	University of Tongren, Chen Shixue	CN105440151A	2016-03-30
8	Ultrasonic-assisted extraction process for pleurotus eryngii polysaccharides	Xiong Derun	Sichuan Yanshan Chinese Herbal Pieces Co. Ltd.	CN103497259A	2014-01-08
9	Ultrasonic-assisted extraction process of macadamia nut polypeptide	Wang Hongsheng	Zhuhai Liangpu Food Co. Ltd.	CN111471730A	2020-07-31
10	Method for ultrasonic-assisted extraction of betanin pigment	Li Junfeng, Ma Chengxiao, Yang Jie, He Xinlin, Yang	University of Shihezi	CN110041727A	2019-07-23

		Yulin, Tao Junhong, Song Dongbao, Teng Xiaolei, Wei Zhen, Chen Cuizhong			
11	Method for preparing instant coffee by ultrasonic-assisted extraction	Tianrui Zhao, Jian Fan	University of Kunming Science & Technology	CN102422959A, CN102422959B	2012-04-25, 2013-11-27
12	Ultrasonic-assisted extraction process of prickly pear seed oil	Bai Changcai, Wang Yi, Han Lu, Li Hangying, Fu Xueyan, Zhang Xiaoyan, Tao Li	University of Ningxia Medical	CN114752438A	2022-07-15
13	Method for ultrasonic-assisted extraction of olive pomace oil	He Dongping, Hu Chuanrong, Zhu Jinyan, Liu Lingyi, Peng Hui	University of Wuhan Polytechnic	CN105038943A	2015-11-11
14	Ultrasonic-assisted extraction process of pumpkin seed protein concentrate	Li Haiwang, Tian Shaojun, Bu Guanhao, Xin Ying, Guo Xingfeng, Mo Chongwen, Huang Yan	University of Henan Technology	CN110521848A	2019-12-03
15	Method for ultrasonic-assisted extraction of sorghum red pigment	Zhang Hairong, Chen Jin'e, Hu Yan	University of Xinzhou Teachers	CN109504123A	2019-03-22
16	Method for ultrasonic-assisted extraction of Caulis spatholobi pigment	Wu Guirong, Lin Ning, Chen Chunlan, Chen Qiujuan, Deng Zhiyong, Xiao Xiaochun, Li Muyun	University of Hezhou	CN105754371A	2016-07-13
17	Method for ultrasonic-assisted extraction of sorghum red pigment	Zhang Hairong, Chen Jin'e, Hu Yan	University of Xinzhou Teachers	CN109504122A	2019-03-22
18	Preparation method for ultrasonic-assisted extraction of sapindus saponin	Qiao Yuping	Nanjing Zhenweikang Biotechnology Co. Ltd.	CN111217884A	2020-06-02
19	Method for ultrasonic-assisted	Ma Haile, Huang Liurong,	University of Jiangsu	CN102850245A, CN102850245B	2013-01-02, 2014-07-30

	extraction of alliin in garlic	He Ronghai, Ren Xiaofeng, Cao Lijuan			
20	Multi-mode ultrasonic-assisted extraction method of arrowhead starch	Fan Minghong, Zheng Jiayu, Li Hongmei, Yang Kangzhen, Han Hua, Xu Hong, Zhu Yuyuan, Zhou Lu, Zhang Bingqing, Wang Jing	Baoying Organic Food Quality Supervision Examination Center	CN109438582A	2019-03-08
21	Method for ultrasonic-assisted extraction of flammulina velutipes polyphenol	Cao Tianxu, Du Ping, Zhou Jiliang, Cheng Benchun, Chen Yu	University of Yangtze Normal	CN115120621A	2022-09-30
22	Method for ultrasonic-assisted extraction of rye bran bound phenol	Chen Xi, Sun Kuijie, Ding Wenping, Wang Yuehui, Lyu Qingyun, Chang Xianhui, Wang Guozhen, Chen Lei, Zhuang Kun, Cao Yang, Zhao Yue	Wuhan Light Industry University	CN114515320A	2022-05-20
23	Method for ultrasonic-assisted extraction of RG-I-rich pectin	Ye Xingqian, Hu Weiwei, Chen Shiguo, Cheng Huan, Liu Donghong	University of Zhejiang	WO2021108979A1	2021-06-10
24	Ultrasonic-assisted extraction method for anthocyanin in hibiscus syriacus petals	Liu Qunlu, Zhang Baozhi, Wang Tingting, Zhang Yanting, Li Xin, Tang Rong	University of Shanghai Jiao Tong	CN103601770A, CN103601770B	2014-02-26, 2016-03-16
25	Ultrasonic-assisted extraction and graded purification method for asparagus saponin	Bing Zhao, Jian Sun, Lingyun Yao, Liwei Wang, Xiaodong Wang, Xiaofan Yuan, Yunxiang Huang	Institute of Process Engineering, Chinese Academy of Sciences	CN102190697A, CN102190697B	2011-09-21, 2013-02-27
26	Sweep frequency ultrasonic-assisted extraction method for alliinase in garlic	Ma Haile, He Ronghai, Huang Liurong, Ren Xiaofeng, Cao Lijuan	University of Jiangsu	CN102851271A, CN102851271B	2013-01-02, 2014-07-30

27	Ultrasonic-assisted extraction device and method for rapidly extracting rhynchophylline	Sun Chao, Zhang JiaChun, Zeng Xianping, Hu Hualin, Zhang Zhenming	The Key Laboratory of Chemistry for Natural Products of Guizhou Province and Chinese Academy of Sciences	CN115583949A	2023-01-10
28	Ultrasonic-assisted extraction technology of Zingiber striolatum free amino acids	Chen Shixue, Yao Yuanyong, Wang Yanyang, He Laibin	University of Tongren	CN110152345A	2019-08-23
29	Response surface method optimized table grape polysaccharide ultrasonic-assisted extraction process	Li Qiu, Huang Xiaoli, Liu Congmin, Leng Xiangpeng, Miao Wenjun	University of Qingdao Agricultural	CN113861304A	2021-12-31
30	Method for ultrasonic-assisted extraction of anthraquinone compounds in morinda officinalis	Wang Lisheng, Zhou Zhongjie, Liang Pengyun, Liu Xu, Luo Xuan, Jiang Jun, Yang Hua, Chen Haiyan, Lin Cuiwu, Wu Lichuan	University of Guangxi	CN108542949A	2018-09-18
31	Sweep-frequency ultrasonic-assisted extraction method based on alliinase in garlic	Li Chengxiu, Zhang Zongfang	Hengfeng Fresh Produce Co. Ltd.	CN112779244A	2021-05-11
32	An ultrasonic-assisted extraction method for effective components of radix bupleuri	Wang Zugang	Qingdao Qingquan Biotechnology Co. Ltd.	CN105726594A	2016-07-06
33	Device and method for extracting algal polysaccharide by ultrasonic-assisted extraction	Liu Zhenfeng, Chen Shiguo, Wang Tingshan, Dai Shengjia, Zhang Zonghua, Ye Xingqian	Zhejiang Yuxiang Biological Science and Technology Co. Ltd.	CN103265645A, CN103265645B	2013-08-28, 2016-01-20
34	Ultrasonic-assisted extraction process of fruit flesh of Nitraria schoberi L.	Yuan Zenghui, Ma Ye, Li Dongdong, Zhao Shujun	Nanjing Vinson Biotech Co. Ltd.	CN110693925A	2020-01-17

35	Ultrasonic-assisted extraction method for health-preserving health-care instant tea	Fu Tianfu, Fu Tianlong, Rao Genghui, Fang Jizhou, Li Minghong, Zhang Siwei, Lin Qishui, Chen Nan	Fujian Chunlun Group Co. Ltd.	CN109601667A	2019-04-12
36	Preparation device for ultrasonic-assisted extraction of natural plant essential oil	Hu Jianhua	Shenzhen Hengyun Biotechnology Co. Ltd.	CN218474906U	2023-02-14
37	Ultrasonic-assisted extraction device and process for macleaya cordata total alkaloid	Zeng Zhaohong, Zhu Caifu	Micolta Bioresource Inc.	CN109260756A, CN109260756B	2019-01-25, 2019-06-28
38	Method for ultrasonic-assisted extraction of indigo and indirubin from folium isatidis	Li Tao, Zhao Guihong, Zhang Nini, Wang Jing, Wang Zhezhi	University of Shaanxi Normal	CN104971081A	2015-10-14
39	Method for producing codonopsis pilosula yellow wine based on ultrasonic-assisted extraction	Zhang Shengtang, Yin Shiping, Ma Yuanzhu	Gansu Wushanchi Rice Wine Co. Ltd.	CN116769554A	2023-09-19
40	Method for ultrasonic-assisted extraction of polyphenol substances from New Zealand spinach	Li Xinxin, Zhang Yujie, Li Xiaoli, Wang Nan, Wang Xin	University of Jilin	CN104906154A, CN104906154B	2015-09-16, 2018-04-03
41	Response surface methodology optimized ultrasonic-assisted extraction process of cabernet gernischt polysaccharide	Leng Xiangpeng, Wang Peipei, Fang Jinggui, Li Qiu, Li Jizhen	University of Qingdao Agricultural	CN113861307A	2021-12-31
42	Ultrasonic-assisted extraction method for polysaccharide extract and pectin extract of okra	Ni Yuanying, Wang Kunli, He Zhengyu, Pang Sicheng, Liu Bing	University of China Agricultural	CN106699917A, CN106699917B	2017-05-24, 2019-04-30
43	Process for ultrasonic-assisted extraction of	Zhou Caibi, Song Lisha, Zhou Caiyuan,	University of Qiannan Normal	CN110151869A	2019-08-23

	flavonoids from camellia chrysantha (Hu) Tuyama	Wang Jiacai, Liu Liming, Shi Yue, Zhou Xin, Yang Lu, Tao Xu	Nationalities, Guizhou Bishu Tech Service Co. Ltd.		
44	Ultrasonic-assisted extraction and membrane separation purification process of gallnut tannic acid	Huang Qian, Zhang Linzhi, Yin Zitao	Hunan Linong Gallnut Industry Development Co. Ltd.	CN110981923A	2020-04-10
45	Response surface methodology optimized ultrasonic-assisted extraction process for solidago decurrens polysaccharide	Hou Ranran, Li Yuting, Yu Xiaohua, Sun Junle, Li Youying, Liu Jie, Ding Chengfeng, Xu Zhiqiang	University of Qingdao Agricultural	CN112724274A	2021-04-30
46	Technology for ultrasonic-assisted extraction of konjac glucomannan through response surface method optimization	Yang Zhengzhou, Mai Xinyun, Xie Xiaona, Huang Bin, Qin Haiqiang	University of Baise	CN107298724A	2017-10-27
47	Nori protein polysaccharide product and counter-current pulse ultrasonic-assisted extraction method thereof	Haile Ma, Wenjuan Qu, Xiaofeng Ren, Jingkun Yan, Ting Wang	University of Jiangsu	CN102432667A	2012-05-02
48	Preparation method for ultrasonic-assisted extraction of phlorizin from leaves of Malus hupehensis	Dong Yan	Nanjing Xiboen Biotechnology Co. Ltd.	CN111196830A	2020-05-26
49	Method for performing ultrasonic-assisted extraction on saponin from eastern Liaoning Chinese aralis carpopodium	Zhang Xiuling, Tian Yaqin, Du Meiling, Liu Xiaocheng, Xu Ruiru, Wu Guomei, Gao Shihan	University of Northeast Agricultural	CN109157555A	2019-01-08
50	Method for carrying out ultrasonic-assisted extraction on crude	Zhao Liyan, Feng Xin, Li Chengjie, Liao Xiaojun, Chen	University of Nanjing Agricultural	CN106632719A	2017-05-10

	polysaccharide of fresh ginger residue	Guitang, Hu Qiuhui			
51	Method for preparing pectin from citrus fruit peel residues by employing ultrasonic-assisted extraction	Ding Zhien, Li Qingnan, Wei Fen, Yan Han, Ding Yu	University of Anhui Agricultural	CN104086667A, CN104086667B	2014-10-08, 2017-03-08
52	Device for ultrasonic-assisted extraction of isoflavone in fermented soybean and recovery of residues	Wu Rina, Wang Weiming, Song Meijun, Shi Haisu, Wu Junrui	University of Shenyang Agricultural, Heilongjiang Traditional Chinese Medicine Science Academy	CN216653464U	2022-06-03
53	Method for ultrasonic-assisted extraction of total flavones in cassia seed serving as Chinese medicine	Qiao Deliang, Zhu Minggui	Qiao Deliang	CN102283888A, CN102283888B	2011-12-21, 2013-07-10
54	Method for quick pretreatment of raw materials of lentinan and ultrasonic-assisted extraction of lentinan	Liu Yuntao, Han Guoquan, Hu Xinjie, Liu Shuliang, Pu Biao	University of Sichuan Agricultural	CN104371035A	2015-02-25
55	Tea concentrated solution or tea beverage and method for preparing tea concentrated solution or tea beverage by coupling ultrasonic-assisted extraction and electrodialysis desalination	Gao Yanxiang, Chen Jinding	University of China Agricultural	CN115211476A	2022-10-21
56	Method for ultrasound-assisted extraction of lappaconitine	Junyi Ma, Ji Zhang, Yanbin Lin, Guiyin Wang, Manting Wang, Baotang Zhao	University of Northwest Normal	CN102617468A	2012-08-01
57	Extraction method of paclitaxel using	Kim Jin Hyun, Ha Geon Soo	Kongju National University Industry-	KR101789409B1, KR20170106660A	2017-09-22, 2017-10-25

	ultrasound-assisted extraction		University Cooperation Foundation		
58	Ultrasound-assisted extraction method of flavonoid compounds in citrus peel	Gu Qing, Li Ping, Yao Xu, Zhou Qingqing, Meng Xia, Zhang Zhongqin	University of Zhejiang Gongshang	CN113209177A, CN113209177B	2021-08-06, 2022-06-28
59	Ultrasound-assisted extraction method for total flavonoids in polytichum commune	Cheng Xiaoxia	University of Xi'an	CN108524554A, CN108524554B	2018-09-14, 2021-04-16
60	Method for ultrasound-assisted extraction of antioxidant polysaccharide power from okra	Ni Yuanying, Wang Kunli, Wen Xin, Li Mo, Wang Yuxiao, Liu Bing, Chen Xiaosong, Han Qianyun, Geng Na, Guo Mengdi	University of China Agricultural	CN106478828A	2017-03-08
61	Ultrasound-assisted extraction method for xanthine oxidase inhibitors in perilla leaves	Li Huizhen, Zhang Zhijun, Wang Yanan, Cui Lixia, Chen Tie	University of North China	CN110840930A	2020-02-28
62	Method for extracting sesame oil by combining ultrasound-assisted extraction and pressing	Yao Zhiyu	Yao Zhiyu	CN108998203A	2018-12-14
63	Soybean protein's ultrasound-assisted extraction device is drawn to high efficiency	Yang Bin, Nie Ming, Tan Yisheng	Jiangxi Yankershop Food Co. Ltd.	CN206228978U	2017-06-09
64	Method for preparing high-purity water-soluble oleuropein through reduced-pressure ultrasound-assisted extraction	Chen Fuwen, Zhao Gouchao, Yao Ruixing, Shi Gaofeng, Wang Guoying	Chen Fuwen	CN104788515A, CN104788515B	2015-07-22, 2020-09-01
65	Method for extracting natural antioxidant from waste tobacco powder by rotary focusing	Xianzhong Zhang, Xingqian Ye, Yujing Sun, Lifen Zhang, Hongjian Gao	University of Zhejiang	CN102491899A	2012-06-13

	ultrasound-assisted extraction				
66	Continuous flow reactor for microwave- and ultrasound-assisted extraction of biologically active compounds from plants	Pătraşcu Mariana, Kumbakisaka Sylviu Amundala Renaud	Pătraşcu Mariana, Kumbakisaka Sylviu Amundala Renaud	RO130896A0	2016-02-26
67	Ultrasound-assisted extraction process of fritillariae thunbergii polysaccharide and health-care product containing fritillariae thunbergii polysaccharide	Zheng Sai, Zhang Xu, Zhou Dannan, Ye Meiliang, Zheng Zhifei, Wu Mingjiang	University of Wenzhou	CN103483459A	2014-01-01
68	Double-frequency ultrasound-assisted extraction method for grape seed extract and application of grape seed extract as lipase inhibitor	Haile Ma, Wenjuan Qu, Xiaofeng Ren, Xiujuan Ren, Qiaorong Yang	University of Jiangsu	CN101822754A, CN101822754B	2010-09-08, 2012-05-23
